# Ratio of 11-desoxy 17-oxosteroids to creatinine in a population screened for breast cancer.

**DOI:** 10.1038/bjc.1979.121

**Published:** 1979-06

**Authors:** J. Poortman, J. van der Smissen, H. J. Collette, F. de Waard

## Abstract

During a population-based screening project for breast cancer, almost 15,000 women aged 50 years and over have provided a 12 h (overnight) sample of urine for research purposes. In 3,789 women the excretion of 11-desoxy-17-oxosteroids (DOOS) and creatinine was measured. Results were analysed in terms of urinary concentrations and of a ratio between DOOS and creatinine. Age had an effect on DOOS, creatinine and their ratio. Body weight and body surface area had an effect on creatinine excretion and therefore on the ratio. The following variables did not have an appreciable effect on the above-mentioned ratio: a family history of breast cancer, parity and age at first pregnancy, menopause and oestrogenic drugs, and parenchymal pattern of the breast as observed on the xeromammogram. Breast cancer was found at first screening in 106 out of 14,697 women. In 100 of these cases DOOS and creatinine were measured. Excretion values expressed as the ratio between the two, allowing for body surface area, did not differ materially from those of 100 age-matched controls. These results lead the authors to the conclusion that the determination of androgen metabolite excretion in women over 50 years of age is of no help in selecting a group at high risk of breast cancer.


Br. J. Cancer (1979) 39, 688

### RATIO OF 11-DESOXY 17-OXOSTEROIDS TO CREATININE IN A POPULATION SCREENED FOR BREAST CANCER

J. POORTMAN, J. VAN DER SMISSEN, H. J. A. COLLETTE AND F. DE WAARD

*From the Preventicon Centre*, the Department of Endocrinology, University Hospital, and the Institute of Social Medicine, University of Utrecht, The Netherlands*

Received 6 November 1978 Accepted 5 February 1979

**Summary.—During a population-based screening project for breast cancer, almost 15,000 women aged 50 years and over have provided a 12 h (overnight) sample of urine for research purposes. In 3,789 women the excretion of 11-desoxy-17-oxosteroids (DOOS) and creatinine was measured. Results were analysed in terms of urinary concentrations and of a ratio between DOOS and creatinine.**

**Age had an effect on DOOS, creatinine and their ratio. Body weight and body surface area had an effect on creatinine excretion and therefore on the ratio.**

**The following variables did not have an appreciable effect on the above-mentioned ratio: a family history of breast cancer, parity and age at first pregnancy, menopause and oestrogenic drugs, and parenchymal pattern of the breast as observed on the xeromammogram.**

**Breast cancer was found at first screening in 106 out of 14,697 women. In 100 of these cases DOOS and creatinine were measured. Excretion values expressed as the ratio between the two, allowing for body surface area, did not differ materially from those of 100 age-matched controls.**

**These results lead the authors to the conclusion that the determination of androgen metabolite excretion in women over 50 years of age is of no help in selecting a group at high risk of breast cancer.**

EXTENSIVE WORK by Bulbrook *et al.* (1962) has drawn attention to the importance of androgen-metabolite excretion in the treatment of advanced breast cancer. These investigators also ventured the hypothesis that abnormal androgen metabolism might be of predictive value in assessing which women are at high risk of this disease. In 1961 they set up a prospective study on the island of Guernsey and collected 24h specimens of urine in over 5000 women. After a period of 10 years they published the findings in 27 women who had developed mammary cancer since the inception of the study, comparing them with a large number of controls. They concluded that low androgen-metabolite excretion might indicate a higher than normal risk of developing the disease (Bulbrook *et al.*, 1971).

On several occasions Bulbrook and co-workers expressed hope that determination of androgen metabolites in the urine might help in defining a high risk group when screening for breast cancer in a population (see Farewell (1977) and Farewell *et al.* (1978)). We took up this point when we designed a large population study for the early detection of breast cancer in the city of Utrecht and its suburbs. Our aim was not only to test the predictiveness of 11-desoxy 17-oxosteroid (DOOS) excretion in respect of breast cancer, but also to explore its possible relationships with known risk factors of this disease.

* Request for reprints to Prof. F. de Waard, Preventicon, Radboudkwartier 261, Utrecht, The Netherlands.

### MATERIAL AND METHODS

The early-detection study was designed after the success of combined physical examination and mammography in women over 50 years of age had been published by Shapiro *et al.* (1971) from the Health Insurance Plan of Greater New York. After having obtained funds from the Prevention Fund, the Ministry of Health and the National Cancer Campaign (Queen Wilhelmina Fund), a team of the University of Utrecht set up a detection centre in the new business and administrative district of the city of Utrecht, just opposite the Central Railway and Bus Station, so that public transport facilities, were met.

With the co-operation of municipal authorities the total female population of Utrecht (and later also the suburban women) was invited street by street. During 1975, 1976 and the first half of 1977 14,697 women aged 50–66* (*i.e.* born 1911–1925) were investigated; the response rate for the first examination was 72%.

The investigation not only had immediate public health aims but also scientific ones. It is our belief that such large-scale undertakings should be used to learn more about the natural history of the disease. Thus the investigation comprised not only physical examination of the breasts and mammography (xeroradiography) but also a questionnaire with a number of items of epidemiological interest and a few somatometric measurements for assessing nutritional status.

In addition we asked the women to bring with them a bottle containing all urine collected from 8 pm on the previous day until after the first urine of the day of screening; 99·2% of women actually complied with our request.

From each urine specimen 2 samples of 250 ml were taken, frozen at —20°C, sealed in plastic bags and stored under a unique identification number at that temperature in a large refrigerating room outside the city. From time to time samples were brought into the small laboratory of the Preventicon Centre for the semi-automated determination of DOOS (by the method described by Rademaker *et al.*, 1976) and creatinine (Cr) (based on an automated Jaffé reaction).

Following a suggestion by Miller *et al.* (1967) we measured total DOOS rather than one or more of the individual DOOSS, such as aetiocholanolone, androsterone or dehydroepi-androsterone.

The rationale of measuring creatinine was the immediate consequence of our decision to limit the urine collection period to about 12 h. It was thought too much of a burden for the women to collect 24h specimens. Thus, the results of DOOS determination were calculated in mg/l and also in terms of a DOOS/Cr ratio.

In order to justify the latter procedure a pilot study was carried out by asking a number of women to provide not only the urine voided between 8 pm and 8 am (overnight urine) but also the following specimens until 8 pm (day urine). The results from this sample of 44 women were as follows:

1. The (Pearson) correlation coefficient between the DOOS/Cr ratio during the night and the excretion of DOOS per 24 h was 0·95.
2. The correlation coefficient between the DOOS/Cr ratio during the night and the excretion of DOOS per 24 h was 0·72. This implies that about half the variation of the latter can be "explained" statistically by the ratio during the night (Fig. 1).
3. The spread in Cr excretion values could not be reduced substantially by intro-

FIG. 1.—Correlation between DOOS/Cr in a 12h overnight specimen of urine and DOOS excretion per 24 h ($r = 0{\cdot}72$).

* The discrepancy between age range and year-of-birth range is due to the fact that it took 2½ years to screen the total cohort of women. In various tables we refer to the oldest group as aged 50–64 years.

ducing parameters of body size or fat-free body mass.

We concluded that the DOOS/Cr ratio in the overnight samples of urine could be used as a measure of androgen-metabolite excretion.

Since the number of urine specimens far exceeded the capacity of our laboratory, the following plan was made.

The intake of women in the study was divided into 5 successive study cohorts numbered I–V. Cohort I had its initial examination during the first half of 1975, Cohort II during the second half of 1975, etc.

In Cohort I all urines (n=2171) were examined. From Cohorts II–IV 2 sets of samples were studied:

1. A randomly selected group (n=790) matched by day of screening, with
2. A compound high-risk group consisting of women with one or more risk factors (n=828) such as previous history of breast biopsy, family history of breast cancer, late age at first pregnancy combined with definite overweight.

Moreover, the 12h urine specimen of 100/106 women found to have mammary cancer through our screening effort could be analysed.

An interesting feature of such a series is that the outcome can be equated more or less to a prospective study, since cancers would have been found later (and in a different time order) if screening had not been carried out. Thus, in a way we had found an economic means of repeating the Guernsey study, though in a somewhat older group of women.

### RESULTS

### *Possible relationships between DOOS/Cr ratio and known risk factors of breast cancer*

*Age.*—The effect of age on DOOS excretion is well known from (*inter alia*) Bulbrook's studies. Within the fairly small age interval of our study a decreasing trend of the DOOS/Cr ratio with increasing age can be seen (Table I). Separate analyses of DOOS and Cr excretion (expressed per litre) show that the androgen-metabolite excretion decreased somewhat faster than Cr excretion with age (Fig. 2). Thus, in analysing the effect of other determinants it has been necessary to control for age.

*Family history of breast cancer.*—Each woman was questioned regarding mastectomy in her mother and/or sister(s). We accepted a history of mastectomy at face

TABLE I.—*Effect of age on ratio of DOOS and creatinine excretion (DOOS/Cr). Cumulative per cent distribution and total number of women investigated in Cohort I.*

| | Age | | |
|---|---|---|---|
| DOOS/Cr | 50–54 (n=1008) | 55–59 (n=687) | 60–64 (n=476) |
| <2 | 3·1 | 4·9 | 5·3 |
| 2–2·9 | 25·4 | 29·4 | 35·6 |
| 3–3·9 | 58·8 | 62·2 | 67·1 |
| 4–4·9 | 81·8 | 83·5 | 88·9 |
| 5–5·9 | 91·5 | 93·7 | 95·8 |
| 6–6·9 | 96·0 | 97·2 | 97·3 |
| ≥7 | 100·0 | 100·0 | 100·0 |

FIG. 2.—Percentages of androgen-metabolite (DOOS) concentration in urine >4 mg/l and of urinary concentration of creatinine >1 g/l according to age. ●, DOOS; ○, Cr.

TABLE II.—*Numbers of women investigated, those with a family history of mastectomy (probands) vs controls*

| | Cohort I | | Random | | "High risk" | | Total | |
|---|---|---|---|---|---|---|---|---|
| Mother mastectomy *vs* controls | Probands | Controls | Probands | Controls | Probands | Controls | Probands | Controls |
| 50–54 | 30 | 939 | 10 | 258 | 75 | 137 | 115 | 1334 |
| 55–59 | 22 | 630 | 11 | 250 | 81 | 110 | 114 | 990 |
| 60–64 | 19 | 429 | 7 | 219 | 53 | 109 | 79 | 757 |
| 50–64 | 71 | 1998 | 28 | 727 | 209 | 356 | 308 | 3081 |
| Sister mastectomy *vs* controls | | | | | | | | |
| 50–54 | 34 | 721 | 8 | 204 | 78 | 96 | 120 | 1021 |
| 55–59 | 35 | 483 | 11 | 200 | 93 | 88 | 139 | 771 |
| 60–64 | 27 | 332 | 11 | 179 | 85 | 92 | 123 | 603 |
| 50–64 | 96 | 1536 | 30 | 583 | 256 | 276 | 382 | 2395 |

Differences in total numbers of controls from total numbers mentioned in the text are due to deliberate omission of women with unknown family history. Differences between controls of "mother series" and controls of "sister series" are due to deliberate omission of those women from the latter series who had no sisters. Control groups have a negative family history of breast cancer for both mothers and sisters.

value, since in a previous study (de Waard *et al.*, 1964) we had found that mastectomy almost always meant breast cancer.

The analysis was carried out on an age-specific basis, separating Cohort I from Cohorts II–IV initially, and later combining them. In studying the distributions of DOOS/Cr ratios the population of women with mastectomized sister(s) was compared with other women having sisters. As controls for those with a history of mastectomy in their mothers, all other women with no such family history (from Cohort I or the random sample of Cohorts II–IV) were used.

FIG. 3.—Cumulative frequency distributions of DOOS/Cr in women aged 50–54 years with a sister or a mother having undergone mastectomy and in controls.

The numbers of women investigated are summarized in Table II, and the results in women aged 50–54 are shown in Fig. 3.

The conclusion is entirely negative, in that we found no differences between those with a family history of mastectomy and controls. The same conclusion was reached for those aged 55 and over.

Since the sample is unbiased and its size is large, we are inclined to believe that this result, which adds the older age range to a smaller study by Bulbrook (1972), is definite.

*Parity and age at first pregnancy.*—In the analysis we have distinguished the nulliparous from the parous. In the former the unmarried have been separated from the ever-married women, and in the parous a division has been made between those who gave birth to their first child before and after 30 years of age respectively. The

TABLE III.—*DOOS/Cr ratio according to marital status, parity and age at first birth, by age. Cross-sections from cumulative frequency distributions (Cohort I and random sample of Cohorts II–IV)*

| Age (yrs) | | No. of women studied | % with DOOS/Cr <3 | % with DOOS/Cr <4 |
|---|---|---|---|---|
| 50–54 | Unmarried | 112 | 26·8 | 57·1 |
| | Married no child | 127 | 23·6 | 55·9 |
| | Age at 1st birth | | | |
| | <30 | 769 | 27·2 | 60·6 |
| | ⩾30 | 271 | 22·1 | 56·8 |
| 55–59 | Unmarried | 75 | 26·7 | 61·3 |
| | Married no child | 87 | 34·5 | 67·8 |
| | Age at 1st birth | | | |
| | <30 | 604 | 32·6 | 64·1 |
| | ⩾30 | 193 | 26·9 | 59·1 |
| 60–64 | Unmarried | 77 | 32·5 | 66·2 |
| | Married no child | 79 | 32·9 | 73·4 |
| | Age at 1st birth | | | |
| | <30 | 387 | 37·2 | 67·7 |
| | ⩾30 | 169 | 39·6 | 68·6 |

results are shown in Table III, in which cross-sections from the cumulative distributions are presented. No clear differences in the DOOS/Cr ratio are apparent.

A more detailed analysis of the effect of age at first birth in Cohort I (Table IV) also shows no clear trend.

*Menopause and oestrogens.*—The analysis has been limited to those under 60 years of age. In the 50–54 age group a substantial proportion of women has not yet reached the menopause (Table V).

TABLE IV.—*Cross-sections from the cumulative frequency distributions; percentage of women according to age at 1st birth (Cohort I) 50–54 years of age, having DOOS/Cr <3 and <4 respectively.*

| Age at 1st birth | No. of women | % with DOOS/Cr <3 | % with DOOS/Cr <4 |
|---|---|---|---|
| <21 | 64 | 25·0 | 60·9 |
| 22–23 | 77 | 24·7 | 49·4 |
| 24–25 | 163 | 19·6 | 62·5 |
| 26–27 | 163 | 29·5 | 58·9 |
| 28–29 | 123 | 35·0 | 65·9 |
| ⩾30 | 207 | 19·8 | 53·6 |
| Total | 797 | | |

TABLE V.—*Effect of menopause and oestrogens. Cross-sections from the cumulative distributions of women (Cohort I) 50–54 and 55–59 years of age, with percentages of women having DOOS/Cr <3 and <4 respectively*

| | No. of women | % with DOOS/Cr <3 | % with DOOS/Cr <4 |
|---|---|---|---|
| Age 50–54 yrs | | | |
| Premenopausal | | | |
| on oestrogenic drugs* | 74 | 28·5 | 60·9 |
| not on oestrogens | 369 | 24·7 | 59·1 |
| Postmenopausal | | | |
| not on oestrogens (menopause natural) | 315 | 24·1 | 57·1 |
| menopause artificial | 161 | 26·1 | 60·9 |
| on oestrogen drugs | 89 | 30·4 | 59·6 |
| Age 55–59 yrs | | | |
| Total cohort | 687 | 29·4 | 62·2 |
| On oestrogenic drugs | 57 | 28·1 | 59·7 |

* Including contraceptive pill.

In the postmenopausal group a distinction between natural and artificial menopause has been made; in those on oestrogens (n=89) the proportion of excretion values (DOOS/Cr ratio) lower than 3 is slightly but not significantly increased, and this trend is not seen in those aged 55–59.

*Weight, height, overweight (Quetelet's index) and body-surface area (as estimated from weight and height).*—In analysing the effect of these variables some conspicuous trends become apparent; *viz.* a tendency to lower values of the DOOS/Cr ratio with increasing body weight (and variables derived from it). This prompted us to analyse these effects (of weight *etc.*) on DOOS and Cr urinary concentrations separately. Neither weight nor height had a clear relationship with the concentration of DOOS in urine; however, they did have an effect on the urinary concentration of Cr. The effect of weight is more marked than that of height. For theoretical reasons (height is correlated with lean body mass, the origin of creatin and its metabolite creatinine) we have preferred to use body-surface area which is derived from both weight and height (Gehan & George, 1970). Fig. 4 shows the effect of this variable.

Therefore, there is a minor effect of

FIG. 4.—Percentages of women with urinary androgen-metabolite concentration (DOOS) >4 mg/l and with urinary creatinine excretion >1 g/l according to body size (body surface area). ●, DOOS; ○ Cr.

body mass and size on the DOOS/Cr ratio. In the analysis of possible differences between cases and controls this will be taken into account.

*Parenchymal pattern of the breast.*—Wolfe (1976) has drawn attention to the existence of parenchymal breast patterns seen on the mammogram which might indicate an increased risk of breast cancer, *viz.*, those with "dysplasia" (DY) and with a prominent duct pattern (PDP). We have (unpublished data) some confirmatory evidence on this point and it seemed logical to look for any relationship between mammographic and endocrine variables.

However, as shown in Table VI, we found no abnormal distribution of the DOOS/Cr ratio in women showing these mammographic images.

### *Case-control comparison*

The prime objective of the study was to test Bulbrook's hypothesis that androgen-metabolite excretion might be of help

TABLE VI.—*DOOS/Cr ratio and parenchymal pattern of breast on (X-ray) xeromammograms (dysplasia=DY; Prominent duct pattern=PDP) in combined women of Cohorts I and II–IV (random group) with no palpable breast lumps. Cross-sections from cumulative frequency distributions.*

| Age (yrs) | Parenchymal pattern of breast on X-ray | No. of woman studied | % with DOOS/Cr <3 | % with DOOS/Cr <4 |
|---|---|---|---|---|
| 50–54 | Normal | 995 | 26·2 | 61·3 |
| | DY | 207 | 23·7 | 58·4 |
| | PDP | 54 | 24·0 | 53·7 |
| 55–59 | Normal | 768 | 31·0 | 63·9 |
| | DY | 141 | 31·9 | 61·7 |
| | PDP | 46 | 32·6 | 58·7 |
| 60–64 | Normal | 556 | 37·2 | 68·0 |
| | DY | 105 | 33·3 | 68·6 |
| | PDP | 42 | 38·1 | 64·3 |

Note: DY and PDP sometimes co-exist. Such mammograms have been counted as both DY and PDP.

in selecting a high-risk group when screening a population for breast cancer.

Since 2 factors were found to have an effect on the DOOS/Cr ratio in a 12h urine (age and body mass or body size), it was decided in the analysis to match for age and to control for body-surface area (as estimated from weight and height).

Thus in our files, for each case of breast cancer found at screening, a matched control was found who did not differ by more than 1 year in age with the case, and who had been screened at about the same time (usually the same day).

Body-surface area was estimated by applying the formula of Gehan & George (1970), viz.:

$$\text{Body Surface Area (BSA)} = 0{\cdot}0235 \times H^{0{\cdot}422} \times W^{0{\cdot}514}$$

where H=height in cm and W=weight in kg.

The results of the comparison are given in Fig. 5 a, b, c for each age group (age at screening) separately. No differences between cases and controls can be found in the DOOS/Cr ratio, taking into account their body-surface area. In the younger age group (50–54 years) there is a slight suggestion for lower ratios in the control group, but this is possibly due to a few control women with large body size (and therefore high Cr excretion).

### DISCUSSION

The present study has produced mainly negative results, in that we have not been able to substantiate Bulbrook's hypothesis on the possible selection of a high-risk group on the basis of androgen metabolite excretion.

It could be argued that our women were somewhat older (50–64 years) than those at Guernsey (35–55 years at entry). Judging from the paper of Bulbrook *et al.*

FIG. 5(a).

FIG. (5b).

FIG. 5(c).

FIG. 5.—Plots of DOOS/Cr *vs* body surface area in breast-cancer cases* (found at screening) and controls (○) matched for age. Cases with lobular carcinoma *in situ* have been underlined in the plot.

(a) Women aged 50–54.
(b) Women aged 55–59.
(c) Women aged 60–64 (60–66 years during the course of the project).

(1971), the latter developed their cancer when they were between 40 and 60 years of age. Since then the number of breast cancers found in Guernsey has increased to 45 (Farewell *et al.*, 1978) and the risk of low androgen levels seems to hold for premenopausal women only (Bulbrook, personal communication).

For logistic reasons, 12h specimens were used in our study instead of 24h specimens. It was shown that the correlations between the DOOS/Cr ratio of 12 h and 24 h specimens are very high ($r = 0{\cdot}95$), whereas this ratio in the 12h overnight sample explains about 50% of the variation of 24 h excretion of DOOS ($r = 0{\cdot}72$).

Creatinine measurements have been introduced as a means of controlling for individual dilution of urine. This introduces a problem in so far as Cr excretion itself is not independent of age and of body mass or size. These variables have been taken into account in comparing cases with controls.

Thus our results are free of this kind of bias.

We are aware of the fact that there may be some day-to-day variation in the excretion of both creatinine (Vestergaard, 1978) and DOOS, although both are said to be fairly constant over time. Data are available on 461 women in whom a second 12 h sample was collected one year later. On the basis of urinary concentrations the correlation coefficient between the first and the second sample was 0·47 for DOOS and 0·38 for Cr. The issue of variability over time, however, is of theoretical rather than of practical interest when trying to identify a high risk group in population screening. Anxiety and stress might influence androgen-metabolite excretion. However, the majority of the Utrecht women screened for breast cancer for the first time in their lives experienced a degree of anxiety. In this respect the women found to have breast cancer were in the same position as the other women who did not have the disease.

Summarizing, we have not been able to find differences in the DOOS/Cr ratio which could be used to identify a group at high risk of breast cancer during a population-screening programme offered to a population of 50 years and over.

This does not imply that androgen metabolism is unrelated to the risk of breast cancer or to the course of the disease. It may be that the predictive value of androgen levels is greater in premenopausal than in postmenopausal women. For the time being, however, breast-cancer screening is almost exclusive to the latter group.

The authors thank Mrs A. Jongejan, Mrs S. Plomp and Miss C. Metsemaekers and Mr B. Rademaker for their support in developing and maintaining the laboratory determinations, Mr B. Slotboom for his help in the data processing and Mrs L. A. Kalff and Mrs J. Bouwman for assistance in preparing the paper.

### REFERENCES

BULBROOK, R. D. (1972) Urinary androgen excretion and the etiology of breast cancer. *J. Natl. Cancer Inst.* **48**, 1039.

BULBROOK, R. D., HAYWARD, J. L., SPICER, C. C. & THOMAS, B. S. (1962) A comparison between the urinary steroid excretion of normal women and women with advanced breast cancer. *Lancet*, ii, 1235.

BULBROOK, R. D. & HAYWARD, J. L. (1969) Excretion of urinary 17-OHCS and 11-desoxy-17-oxosteroids by women using steroidal contraceptives. *Lancet*, ii, 1033.

BULBROOK, R. D., HAYWARD, J. L. & SPICER, C. C. (1971) Relation between urinary androgen and corticoid excretion and subsequent breast cancer. *Lancet*, ii, 395.

DE WAARD, F., BAANDERS-VAN HALEWYN, E. A. & HUIZINGA, J. (1964) The bimodal age distribution of patients with mammary carcinoma, *Cancer*, **17**, 141.

FAREWELL, V. T. (1977) The combined effect of breast cancer risk factors. *Cancer*, **40**, 931.

FAREWELL, V. T., BULBROOK, R. D. & HAYWARD, J. L. (1978) Risk factors in breast cancer: a prospective study in the island of Guernsey. In *Early Diagnosis of Breast Cancer, Methods and Results*. Ed. E. Grundmann & L. Beck. Stuttgart: Gustav Fischer Verlag. p. 43.

GEHAN, E. A. & GEORGE, S. L. (1970) Estimation of human body surface area from height and weight. *Cancer Chemother. Rep.*, **54**, 225.

MILLER, H., DURANT, J. A., JACOBS, A. G. & ALLISON, J. F. (1967) Alternative discriminating function for determining hormone dependency of breast cancer. *Br. Med. J.*, i, 147.

RADEMAKER, B., JONGEJAN-TIMMER, A., POORTMAN, J. & THIJSSEN, J. H. H. (1976) A semi-automated method for the determination of 11-desoxy-17-oxosteroids in urine. *Clin. Chim Acta*, **70**, 349.

SHAPIRO, S., STRAX, P. & VENET, L. (1971) Periodic breast cancer screening in reducing mortality from breast cancer. *J. Am. Med. Ass.*, **215**, 1777.

VESTERGAARD, P. (1978) Excretion of common neutral steroids in healthy subjects as estimated by multi-column chromatography. *Acta Endocrinol.* (*Kbh*) (Suppl. 217), **88**, 157.

WOLFE, J. N. (1976).Risk for breast cancer development determined by mammographic parenchymal pattern. *Cancer* **37** 2486.


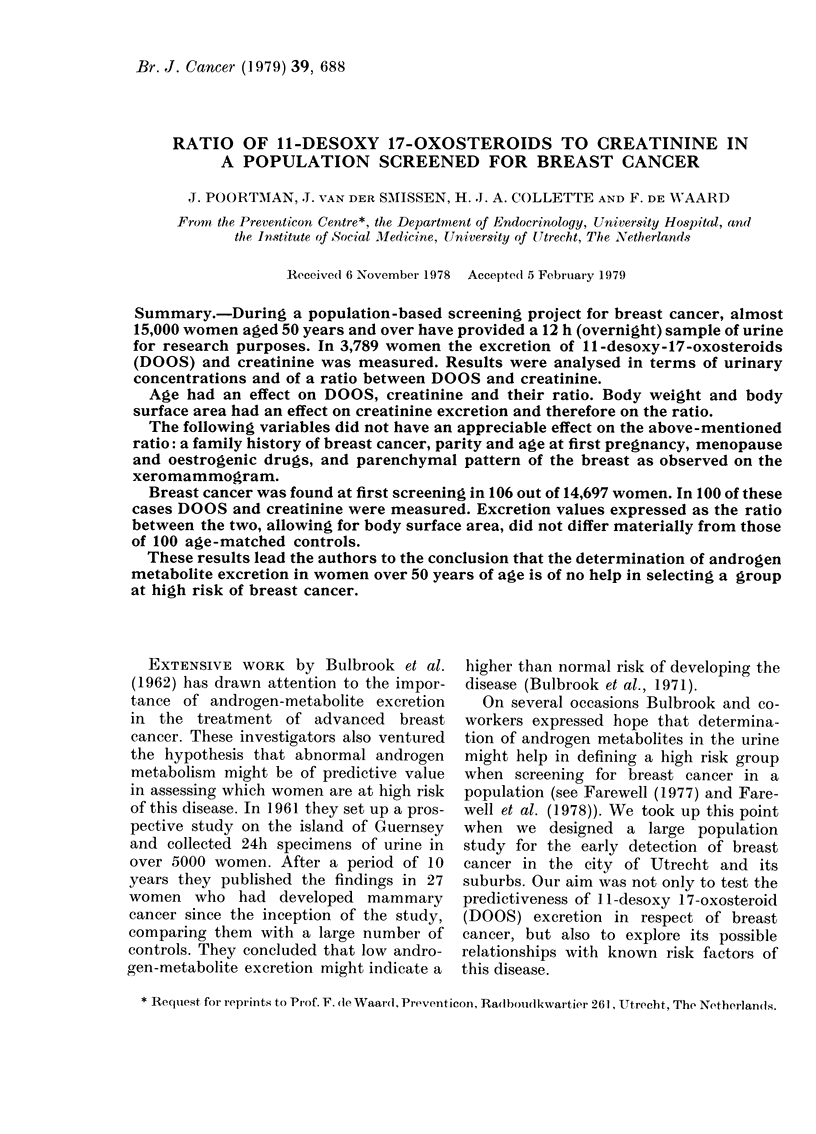

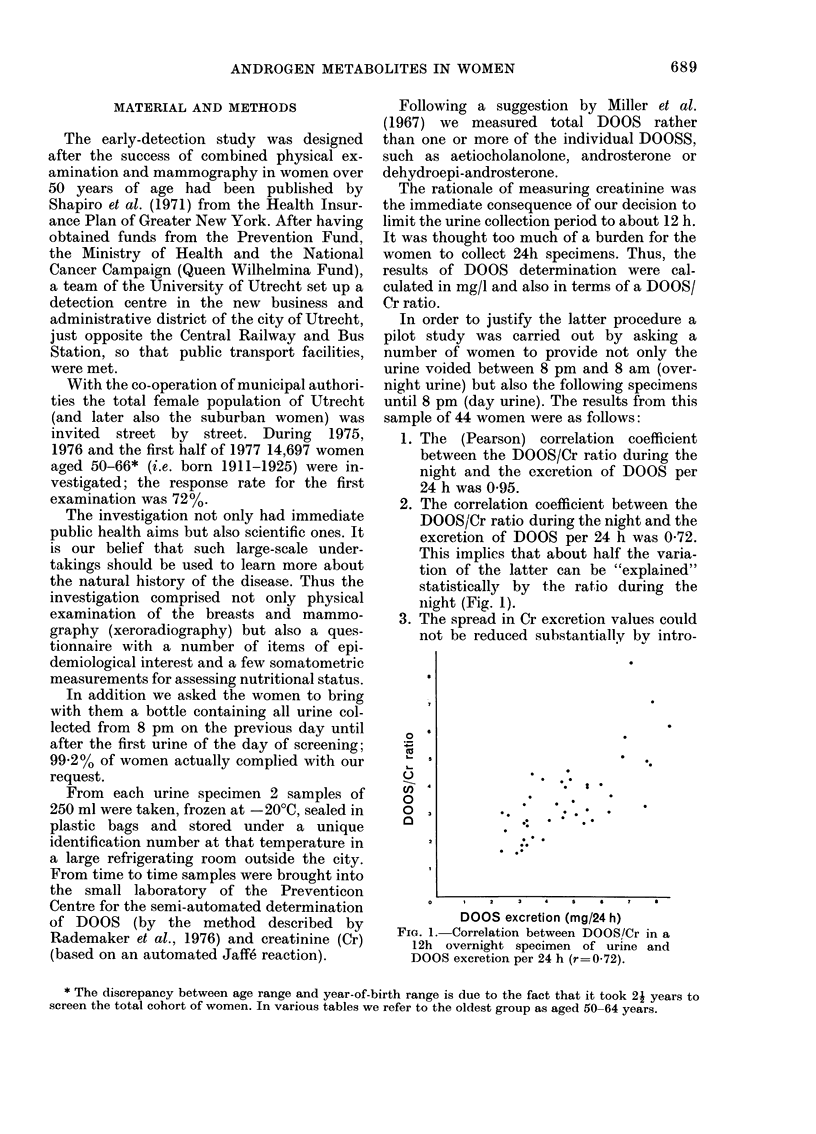

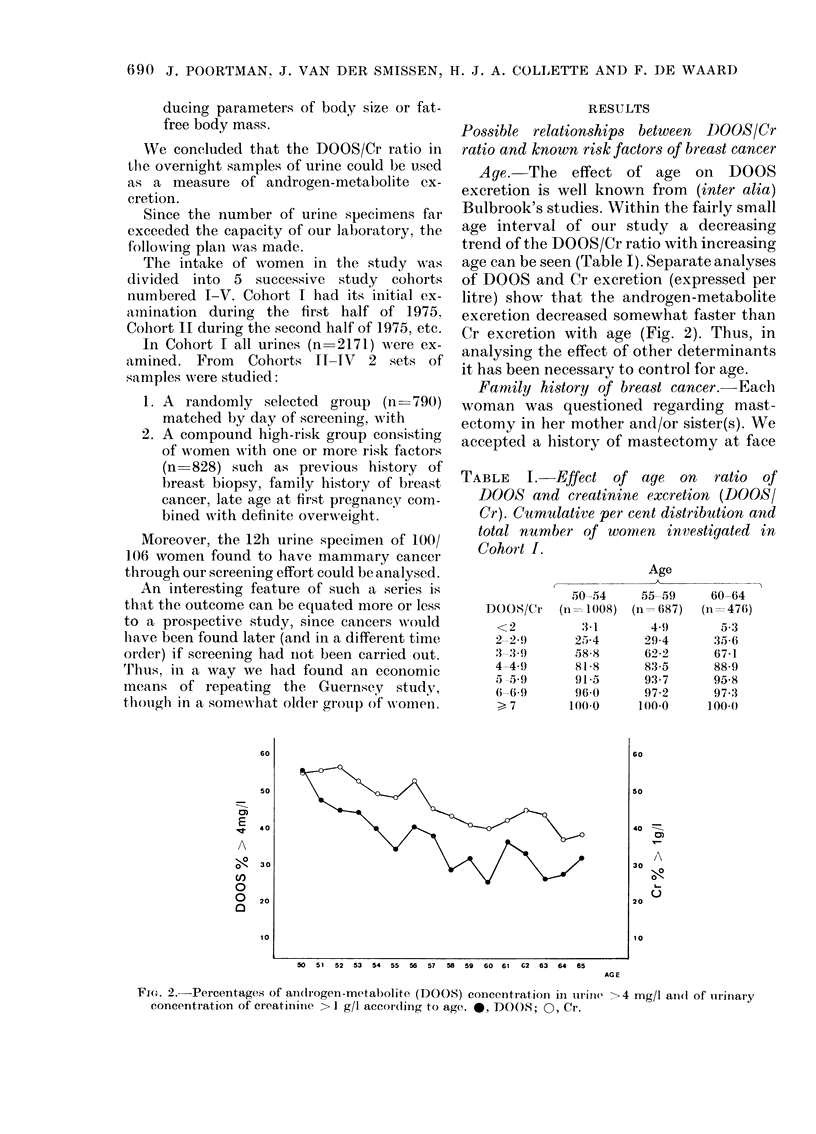

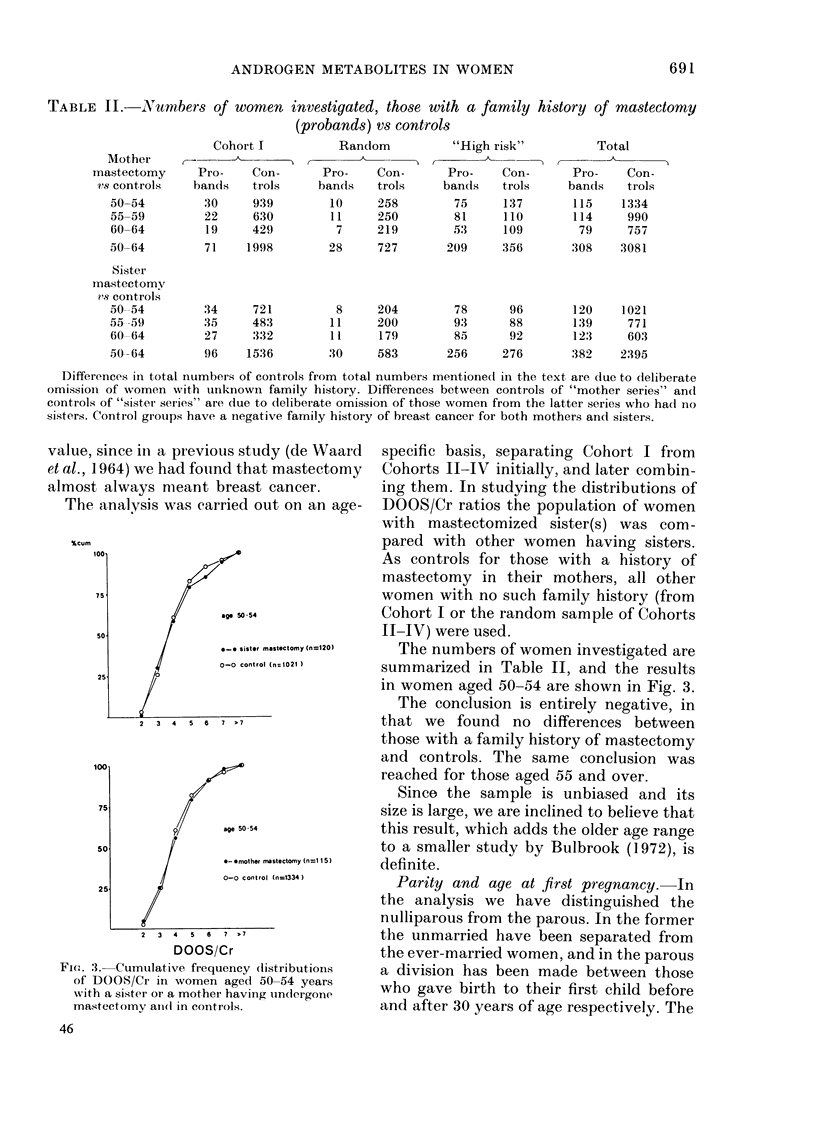

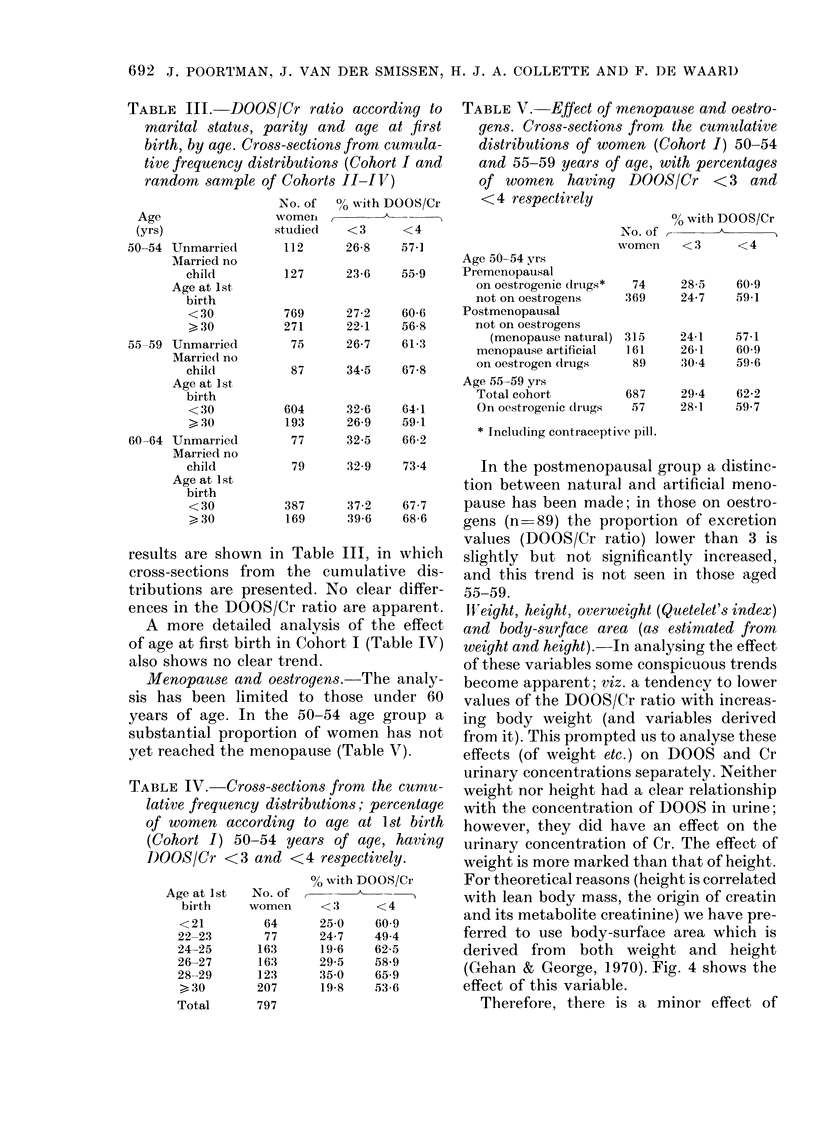

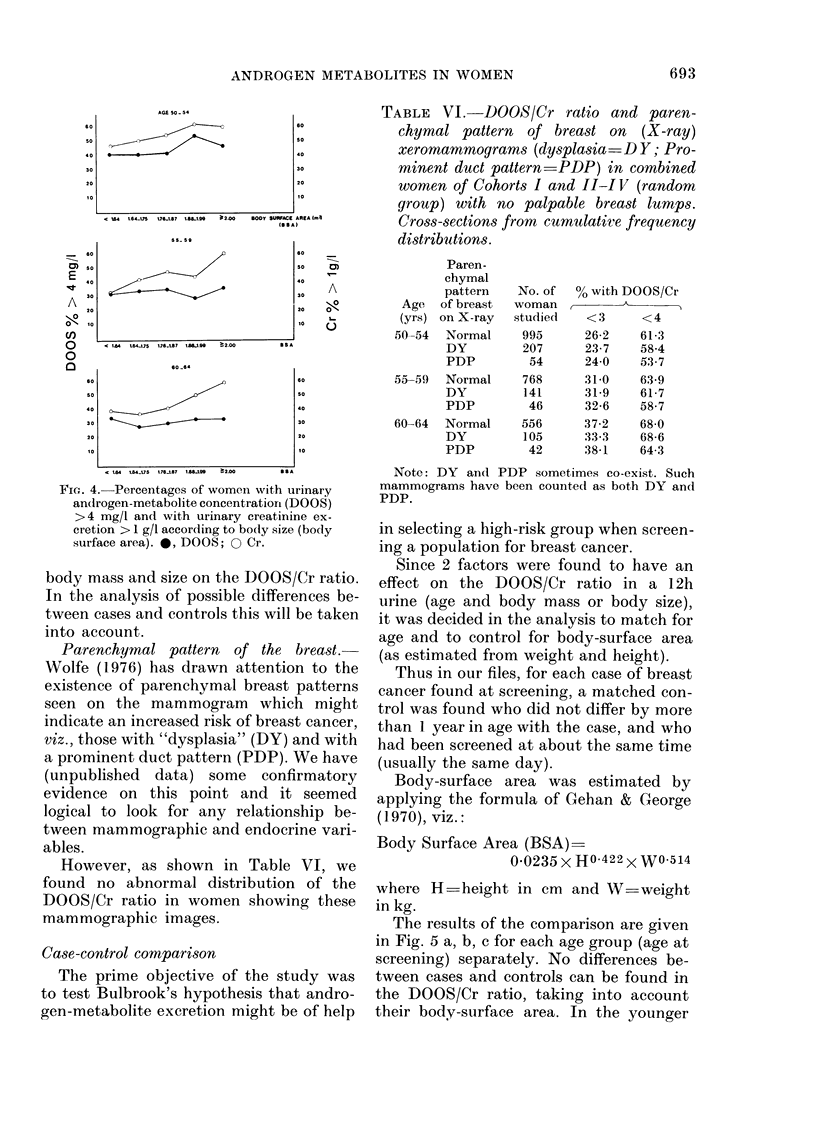

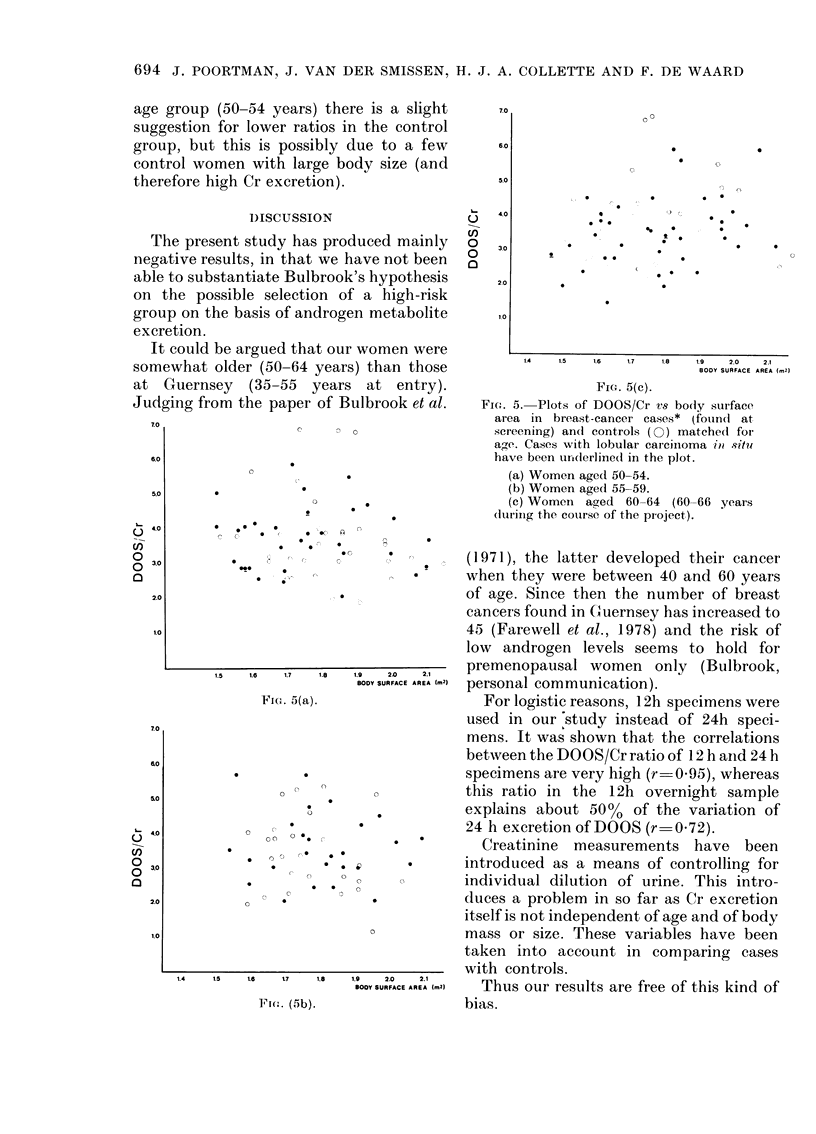

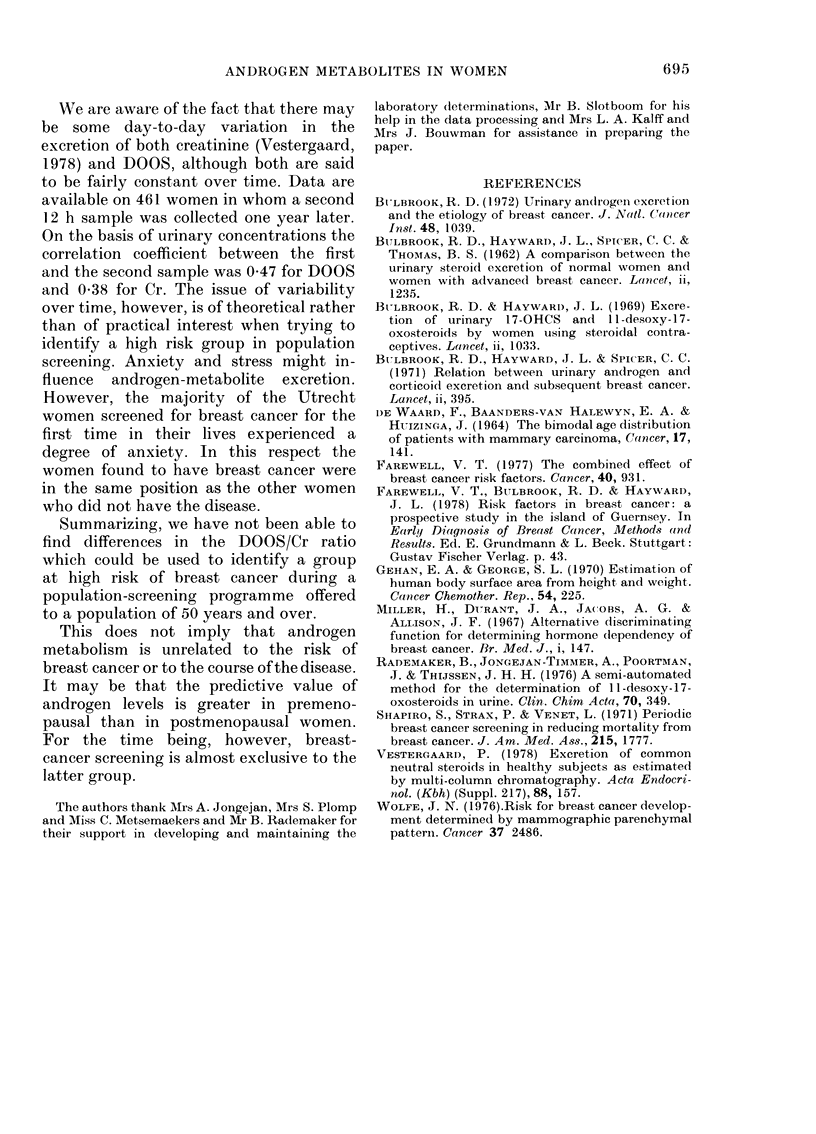


## References

[OCR_01140] BULBROOK R. D., HAYWARD J. L., SPICER C. C., THOMAS B. S. (1962). A comparison between the urinary steroid excretion of normal women and women with advanced breast cancer.. Lancet.

[OCR_01147] Bulbrook R. D., Hayward J. L. (1969). Excretion of urinary 17-hydroxycorticosteroids and 11-deoxy-17-oxosteroids by women using steroidal contraceptives.. Lancet.

[OCR_01153] Bulbrook R. D., Hayward J. L., Spicer C. C. (1971). Relation between urinary androgen and corticoid excretion and subsequent breast cancer.. Lancet.

[OCR_01159] DE WAARD F., BAANDERS-VANHALEWIJN E. A., HUIZINGA J. (1964). THE BIMODAL AGE DISTRIBUTION OF PATIENTS WITH MAMMARY CARCINOMA; EVIDENCE FOR THE EXISTENCE OF 2 TYPES OF HUMAN BREAST CANCER.. Cancer.

[OCR_01165] Farewell V. T. (1977). The combined effect of breast cancer risk factors.. Cancer.

[OCR_01177] Gehan E. A., George S. L. (1970). Estimation of human body surface area from height and weight.. Cancer Chemother Rep.

[OCR_01184] Miller H., Durant J. A., Jacobs A. G., Allison J. F. (1967). Alternative discriminating function for determining hormone dependency of breast cancer.. Br Med J.

[OCR_01188] Rademaker B., Jongejan-Timmer A., Poortman J., Thijssen J. H. (1976). A semiautomated method for the determination of 11-deoxy-17-oxo-steroids in urine.. Clin Chim Acta.

[OCR_01194] Shapiro S., Strax P., Venet L. (1971). Periodic breast cancer screening in reducing mortality from breast cancer.. JAMA.

[OCR_01199] Vestergaard P. (1978). Excretion of common neutral steroids in healthy subjects as estimated by multi-column chromatography.. Acta Endocrinol Suppl (Copenh).

[OCR_01205] Wolfe J. N. (1976). Risk for breast cancer development determined by mammographic parenchymal pattern.. Cancer.

